# Magnetic fields as a potential therapy for diabetic wounds based on animal experiments and clinical trials

**DOI:** 10.1111/cpr.12982

**Published:** 2021-02-08

**Authors:** Huanhuan Lv, Junyu Liu, Chenxiao Zhen, Yijia Wang, Yunpeng Wei, Weihao Ren, Peng Shang

**Affiliations:** ^1^ School of Life Sciences Northwestern Polytechnical University Xi’an China; ^2^ Heye Health Technology Co., Ltd. Anji Zhejiang China; ^3^ Research & Development Institute Northwestern Polytechnical University Shenzhen China; ^4^ Key Laboratory for Space Bioscience and Biotechnology Northwestern Polytechnical University Xi’an China

**Keywords:** diabetic foot ulcers, diabetic wounds, dynamic magnetic field, magnetic fields, static magnetic field, wound healing

## Abstract

Diabetes mellitus (DM) is a chronic metabolic disorder with various complications that poses a huge worldwide healthcare burden. Wounds in diabetes, especially diabetic foot ulcers (DFUs), are difficult to manage, often leading to prolonged wound repair and even amputation. Wound management in people with diabetes is an extremely clinical and social concern. Nowadays, physical interventions gain much attention and have been widely developed in the fields of tissue regeneration and wound healing. Magnetic fields (MFs)‐based devices are translated into clinical practice for the treatment of bone diseases and neurodegenerative disorder. This review attempts to give insight into the mechanisms and applications of MFs in wound care, especially in improving the healing outcomes of diabetic wounds. First, we discuss the pathological conditions associated with chronic diabetic wounds. Next, the mechanisms involved in MFs’ effects on wounds are explored. At last, studies and reports regarding the effects of MFs on diabetic wounds from both animal experiments and clinical trials are reviewed. MFs exhibit great potential in promoting wound healing and have been practised in the management of diabetic wounds. Further studies on the exact mechanism of MFs on diabetic wounds and the development of suitable MF‐based devices could lead to their increased applications into clinical practice.

## Graphical abstract

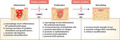

## 1 INTRODUCTION

Skin, the largest organ in human body, has important immune and protective traits also with amazing ability to self‐repair.^1^, ^2^, ^3^ After injury, multiple biological pathways become activated resulting in re‐establishment of tissue integrity. Based on the time required for healing, wounds can be classified into acute and chronic wounds. Acute wounds can be repaired by themselves through the normal healing process resulting in the functional restoration; chronic wounds cannot be repaired through the normal and timely way resulting in prolonged or incomplete repair.^4^ Normal wound repair follows coordinated sequence of haemostasis phase, inflammation phase, proliferation phase and remodelling phase involving crosstalk between different cells, extracellular matrix (ECM) and cytokines in time and spatial dimensions.^5^, ^6^ Chronic wounds develop when there are disruptions in normal healing process and are big challenges to people with diabetes.^7^

In diabetes mellitus (DM), chronic wounds are common on the lower extremities, particular happening at foot, it is so‐called diabetic foot ulcer (DFU).^8^, ^9^ Development of chronic wounds in people with diabetes possibly results in high risk of limb amputation if they are not treated effectively.^10^ Diabetic wounds are hard to care and manage in clinics. Development of more effective managements for diabetic wounds is urgent and imperative. Many efforts and studies have been focused on wound care with an emphasis on the physical approaches and the development of related device for enhancing the healing rate of diabetic wounds.^11^, ^12^

Over the history, people have explored magnetic fields (MF) from not only nature but also artificial sources for therapeutic uses. Nowadays, MF has been developed as an alternative, noninvasive and safe therapeutic tool for tissue repair due to the beneficial effects on cell migration, proliferation and adhesion.^13^, ^14^, ^15^, ^16^, ^17^, ^18^ In spite of MFs’ potential for their therapeutic application, the safety of MFs including static magnetic field (SMF), extremely low‐frequency electromagnetic field (ELF‐EMF) and pulsed electromagnetic field (PEMF) is still widely discussed and considered. International Commission on Non‐Ionizing Radiation Protection (ICNIRP) demonstrates that no evidence supports the adverse effect on human after exposure to up to 8 T SMF.^19^ There is no adverse effect to experimental mice that are short‐termly or long‐termly exposed to high or even ultrahigh SMF.^20^, ^21^, ^22^ At present, it reaches no exact conclusion about the adverse impact of dynamic MFs on human body, moreover, dynamic MFs are widely used in clinics for treatment of bone diseases and neurodegenerative and related disorder.^23^, ^24^, ^25^, ^26^

MFs affect cellular function and activities by their actions of electric/magnetic properties or magnetic property alone on cellular processes and functional molecules and act as a kind of potential therapy for wound repair as far as wounds in DM.^16^, ^17^, ^27^, ^28^, ^29^ The present review focuses on the pathological conditions associated with chronic wounds in DM. Then, the possible mechanisms involved in MFs’ effects on wound healing are explored. Last, we review the therapeutic effects of MFs on diabetic wounds both from animal experiments and clinical studies and attempt to arouse the interest of pushing forward the applications of MFs on wound healing in DM.

## 2 IMPAIRED WOUND HEALING IN DIABETIC MELLITUS

**FIGURE 1:**
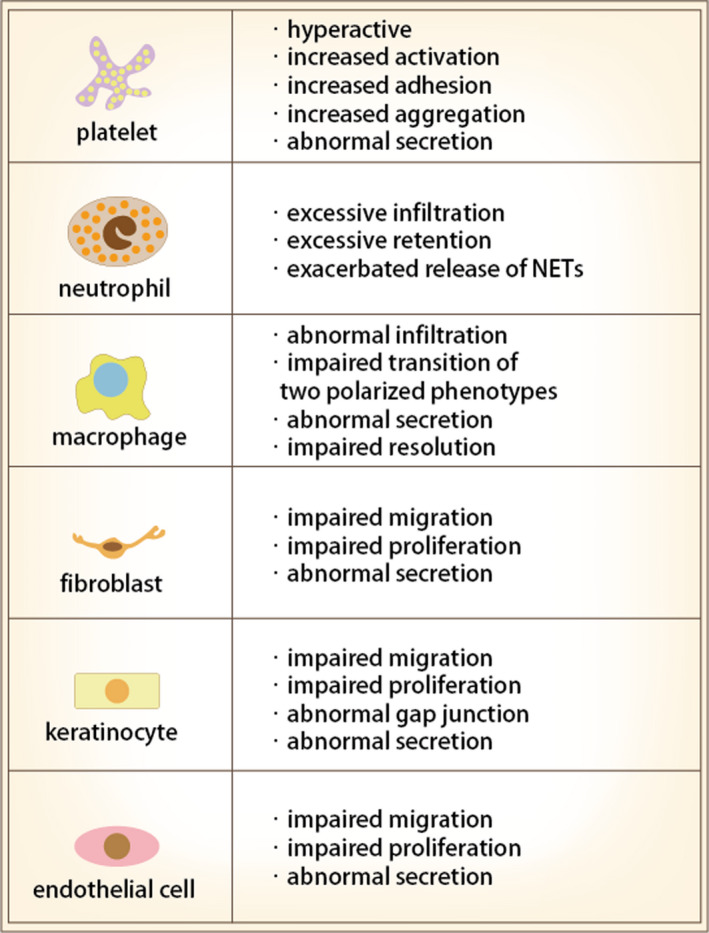
Diabetic condition disturbs the normal activities of cells participate in wound healing. Abbreviations: NET, neutrophil extracellular trap

DM is characterized by hyperglycaemia which is a significant cause in the development of inflammation in diabetic complications.^30^ Hyperglycaemic condition and oxidative burden cause modifications and dysfunctions to cells that participate in wound repair and promote inflammation resulting in inhibitory effects on wound healing.^31^, ^32^ Oxidative stress in DM may generate from glucose metabolism and auto‐oxidation or through the formation of reactive oxygen species (ROS) and advanced glycation end products (AGEs). A state of persistent hyper glycaemic condition the delay of wound healing in DM and promotes the development of chronic wounds.^33^ Wound healing is a highly coordinated biological process.^34^, ^35^, ^36^ After injury, various types of cells, such as platelets, neutrophils, macrophages, fibroblasts, keratinocytes and endothelial cells migrate to wounds to initiate and regulate the repair process. Intrinsic abnormalities and pathological factors in DM disturb the normal activities of cells participate in wound healing and affect their secretions and the communication network which further interrupts the coordinated cascade of events in wound repair process^37^ (Figure 1). Impaired wound healing is commonly encountered in people with diabetes and leads to severely unfavourable outcomes.^32^

### 2.1 DM affects the function of platelets in wound healing

Platelets initiate the earliest events after injury and form a platelet plug in haemostasis phase. Platelet‐derived growth factor (PDGF) stimulates the migration of cells to wounds in inflammation phase, enhances the proliferation of fibroblasts and production of ECM in proliferation phase and regulates matrix metalloproteinases (MMPs) in remodelling phase.^38^ The abnormalities of platelets in DM are characterized to be hyperactive with increased autophagy, activation, adhesion and aggregation.^39^, ^40^, ^41^, ^42^, ^43^ These abnormalities in platelets lead to wound healing dysfunction. Strategies using functional platelets and the combination of their secretions exhibit significant outcomes in managing wounds in DM.^44^, ^45^

### 2.2 DM affects the function of neutrophils in wound healing

Neutrophils are mobilized to inflammatory site upon injury and involved in the early stage of inflammation phase to form a web‐like structure called neutrophil extracellular traps (NETs).^46^, ^47^, ^48^, ^49^, ^50^, ^51^ After completing their function, neutrophils must be eliminated or migrate away within a defined time period. Otherwise, excessive infiltration and retention of neutrophils lead to delayed wound healing.^52^, ^53^ The expressions of microRNA in neutrophils involved in inflammatory response are changed in DM.^54^ DM condition increases the release of NETs through facilitating NETosis, and the exacerbated release of NETs is a key factor for the delayed wound healing.^55^, ^56^, ^57^ Inhibition of NETosis by using gonadotropin‐releasing hormone (GnRH) antagonist improves the delayed wound healing in diabetic mice.^58^

### 2.3 DM affects the function of macrophage in wound healing

Macrophages affect the whole wound healing process by displaying different polarization phenotypes.^59^ The events associated with the end of inflammation phase to the start of proliferation phase are the removal of macrophages by lymphatics and the transition of macrophage polarization from M1 to M2 phenotype.^60^ The hyperglycaemic environment imprints epigenetic modulation in macrophages towards a pro‐inflammatory phenotype which fails to transit to the anti‐inflammatory state.^31^ AGEs modulate macrophage polarization to M1 phenotype which impairs wound healing in DM.^61^ The imbalance transition between two functional phenotypes of macrophage induces the delayed healing process in diabetic wounds.^62^ Treatments with macrophage‐secreting cytokine transforming growth factor (TGF‐β1) or macrophage‐derived exosomes accelerate diabetic wound healing.^63^, ^64^

### 2.4 DM affects the function of fibroblasts in wound healing

Fibroblasts are responsible for producing new ECM and releasing growth factors in wound repair process. Delayed healing in diabetic wounds is attributed to decreased growth rate of dermal tissue which suggests the dysfunctions in fibroblasts.^65^, ^66^ High glucose impairs the migration and proliferation of human gingival fibroblasts by inducing oxidative stress and apoptosis.^67^, ^68^ Excessive accumulations of AGEs contribute to the development of chronic wound healing through inducing autophagic cell death in fibroblasts.^69^ Human skin fibroblasts in people with diabetes exhibit accelerated senescence than the aged‐matched ones from normal volunteers.^70^ Fibroblasts from the wound edges of human DFUs exhibit abnormally high expression of connexin protein which may elevate the gap junctional communication and retard the proliferation of fibroblasts.^71^ Some anti‐diabetic drugs or healthy human fibroblasts or their exosomes have been proved to improve wound healing in DM.^72^, ^73^, ^74^, ^75^

### 2.5 DM affects the function of keratinocytes in wound healing

Keratinocyte is the major cell type of epidermis, its migration and proliferation are important for re‐epithelialization in wound healing process.^76^ Under diabetic condition, keratinocytes exhibit reduced proliferation potential, less migration capacity, abnormal gap junction and expression of MMPs.^77^, ^78^, ^79^ Diabetic condition reduces keratinocytes migration through indirectly changing the activity of macrophage and creating a micro‐environment with high level of tumour necrosis factor α (TNF‐α).^80^ Human keratinocyte‐derived micro‐vesicle that expresses miR‐21 mimic promotes fibroblasts migration, differentiation, contraction and induce a pro‐inflammatory response.^81^

### 2.6 DM affects the function of endothelial cells in wound healing

Vasculogenesis occurs at the late stage in wound healing process. Endothelial cells are responsible for the development of new vessels. Oxidative stress induced by hyperglycaemia causes damage to endothelial cells which further leads to endothelial dysfunction which is implicated as an underlying deterrent to diabetic wound healing.^82^, ^83^, ^84^ The migration pathway of endothelial progenitor cells from bone marrow to the diabetic wounds is dysfunctional.^84^, ^85^ In diabetic condition, phagocytes activated by inflammatory cytokines inhibit the migration and recruitment of endothelial progenitor cells to the wound sites.^86^ The ways to increase the migration and proliferation of endothelial progenitor cells by using platelet‐rich plasma or anti‐diabetic drug promote diabetic wound healing.^87^, ^88^

## 3 THE EFFECTS OF MAGNETIC FIELDS ON WOUNDS

Based on the intensity and direction, MFs are classified as static magnetic field (SMF) and dynamic MF. The intensity and direction are both constant in SMFs, while they are varied with time in dynamic MFs. Studies have proved that MFs improve cell migration and adhesion which could be extremely beneficial to tissue regeneration and wound healing.^28^, ^29^ As a result, MFs, especially dynamic MFs, have been widely developed as an effectively therapeutic tool for repairing tissues.

### 3.1 The effects of SMFs on wound healing

SMF is generated by permanent magnets or by passing direct current through a coil. There is no electric energy in SMF, therefore there is no heat and electrical harm to the tissues. It has been shown great potential in the field of tissue regeneration.^89^ SMF assists in wound healing through promoting cell migration by affecting cellular orientation, morphology and migration.^89^ Different intensities (10, 50, 80 and 100 mT) of SMFs cause no effect on cell viability and no damage on cell membrane of mouse embryonic fibroblasts; however, cell morphology becomes elongated with protrusions and short microvilli possibly as a result of re‐arrangement of cytoskeleton.^90^ The field orientation of SMF affects the bio‐effects differently when applied to wound healing.^91^, ^92^ Exposure to SMF of perpendicular other than parallel direction to the incision increases the strength of cutaneous wounds that are closed primarily.^93^ Wound tissues that exposed to the North pole of SMF show better healing outcome.^94^ Also, local exposure to 180 mT SMF with two attracted neodymium magnets encourages fast wound healing.^95^ Exposure to SMF of 120 μT increases proliferation potential and upregulates endothelial nitric oxide synthase (eNOS) expression in human umbilical vein endothelial cells.^96^ A double‐blind placebo‐controlled pilot study also demonstrates that application of SMF device promotes leg ulcer healing.^97^ Contrasting studies, however, suggest that there are no differences between gross healing parameters, mechanical strength and hydroxyproline deposition regarding wound healing processes in rat with a magnet in contact with wound or not.^98^

### 3.2 The effects of dynamic MFs on wound healing

Dynamic MFs are also able to affect cell morphology, differentiation and function.^28^, ^99^ As to their applications in wound healing, it mainly includes ELF‐EMF and PEMF.^100^, ^101^

ELF‐EMF represents a form of non‐ionizing and low energy radiation with frequency induce a variety of biological effects.^101^ Several studies demonstrate that ELF‐EMF exhibits driving actions on the progression of wound healing. On one hand, ELF‐EMF modulates cytokine profile which drives transition from chronic pro‐inflammatory state to anti‐inflammatory state in wound healing process.^102^ On the other hand, exposure to ELF‐EMF also drives a shift in wound healing process from inflammation phase to proliferation phase.^103^ ELF‐EMF exposure enhances the proliferation of keratinocyte HaCaT cells and improves early NOS activity, while decreases cyclooxygenase 2 (COX‐2) which indicates its role in accelerating the transition from inflammation phase to remodelling phase.^104^ These results hint that ELF‐EMF may play different roles in different phases of wound healing and promote the progression of wound healing.

Moreover, ELF‐EMF has been shown to alter the function of other participants in wound healing. Exposure to ELF‐EMF with frequency of 50 Hz and intensity of 1 mT increases cytokine release and activates the expression of MMP‐9 in human immortalized keratinocytes.^105^ The upregulation of MMP‐9 represents the effect of ELF‐EMF on promoting cell migration and inducing phagocytosis in inflammation phase of wound healing.^106^ ELF‐EMF increases cellular ROS production in human keratinocyte cell line NCTC 2544.^107^ On the contrary, ELF‐EMF activates glutathione peroxidase with decrease in malondialdehyde in the live tissue of rats during wound healing process.^108^ ELF‐EMF promotes the proliferation and differentiation of transplanted epidermal stem cells in the full‐thickness defect nude mice with more mature generated skins and viable cell layers and rich hair follicles’ structure at the wound sites.^109^ ELF‐EMF also directly acts on the ion channel to affect cellular function. Exposure to 50 Hz ELF‐EMF activates macrophage/monocyte through regulating Ca^2+^ ion channel.^110^ After being exposed to ELF‐EMF, the morphology of macrophages change to elongated shape, because the cluster of cation channel receptor alters Ca^2+^ homeostasis and further affects actin polymerization.^111^

PEMF is a kind of low‐frequency magnetic field with specific wave shape and amplitude.^18^ PEMF exposure decreases the production of interleukin‐8 (IL‐8), chemoattractant protein‐1 (MCP‐1) and macrophage inflammatory protein‐1α (MIP‐1α) in human keratinocyte cell line HaCat.^112^ Short‐term exposure to PEMF enhances the re‐epithelialization process and decreases the contraction area at the early stage of wound healing.^113^ Short duration of PEMF exposure accelerates wound healing in a rat wound model through promoting the appearance of loose connective tissue, forming capillaries, increasing re‐epithelization and improving the structure of newly formed collagen fibres.^114^ In a 3D artificial skin stimulated model, PEMF treatment stimulates the early formation of connective tissue, vascular network and collagen synthesis by inducing cell proliferation as well as increasing the adhesion ability and paracrine activity of fibroblasts.^115^ PEMF also increases tensile strength at an early phase of wound healing, but there is no significant increase over time as wounds in the PEMF‐treated group and sham group both reach the maximum mechanical strength at the late phase of wound healing.^116^ PEMF shortens the time for bridging the gap through increasing the proliferation of patellar tendon fibroblasts in an in vitro wound healing experiment.^117^

## 4 MAGNETIC FIELDS PROMOTE DIABETIC WOUND HEALING

After being exposed to the actions of MFs, cellular participants, cytokines and ion channel exhibit alterations in their performance in wound healing process.^100^ MFs promote the progression of wound healing by driving the timely transitions of wound healing stages from pro‐inflammation state to anti‐inflammation state or from proliferation state to remodelling state. MFs exert positive effects on the functions of various cell types that participate in wound healing by promoting their migration, proliferation and regulating their secretory activities. Except the enhanced effects on cellular functions, MFs also improve the mechanical strength of newly formed skin tissue. MFs enhance wound healing process due to its role for generating a favourable environment for tissue repair through stimulating the production of cytokine, increasing cell proliferation and enhancing collagen formation. In diabetic wounds, normal repair process is impaired or dysfunctional. It wonders whether MFs affect the impaired communications and enhance the stagnant stage in diabetic wounds or not. How do MFs affect wound healing process in diabetic environment? The effects of MFs on diabetic wounds from both animal experiments and clinical trials are concluded in Tables 1, 2, 3.

**TABLE 1:** The effects of SMFs on diabetic wound healing based on animal experiments

MF types	Intensity	Exposure manner	Exposure duration	Animal strain	Diabetic model	Wound size	Basic index	Wound‐related indexes	Ref
SMF	180 mT	Local exposure, a magnet with the North magnetic pole oriented towards the dressing	5, 12 or 19 days	Male Sprague‐Dawley rats, 280 g	STZ at 60 mg/kg body weight, 2 weeks after STZ injection	An open circular wound with 15 mm on the dorsum covered with a hydrocolloid occlusive dressing	No change on serum glucose	Improve healing rate; reduce gross healing time; higher tensile strength	^118^
SMF	0.6 T	Whole‐body exposure, a magnetic plate with 24 magnetic pieces	3, 7 or 14 days	Male BKS‐Lepr em2Cd479/Nij (db/db) mice	/	Two excisional wounds on each side of the dorsum with self‐adhesive dressings	/	Reduce wound size; improve wound closure rate; lower the distance between the epithelial tips of punched wound and distances between the edges of the panniculus carnosus	^119^
SMF	230 mT	Local exposure, a magnetic disk with the North magnetic pole oriented towards the gauzes	7, 14 or 21 days	Male Wistar rats	STZ at 65 mg/kg body weight, 3 days after STZ injection	A square full‐thickness incision (2 cm × 2 cm) on the back covered with sterile gauze	No change on blood glucose	Reduce wound area; increase wound mechanical strength; reduce wound closure time	^120^

**TABLE 2:** The effects of dynamic MFs on diabetic wounds based on animal experiments

MF types	Intensity / frequency	Exposure manner	Exposure duration	Animal strain	Diabetic model	Wound size	Basic index	Wound‐related indexes	Ref
PEMF	Commercially available bone‐healing device (EBI) with frequency at 15 Hz, intensity of 12G and pulse width of 4.5 ms	Whole‐body exposure	8 h/day, 7 or 14 days	Db/db C57BL6 mice, 10‐12 weeks	/	A circular full‐thickness wound (5 mm) on the dorsum with a ring‐shape silicone splint and transparent sterile occlusive dressing	/	Improve wound closure rate; increase fibroblast growth factor 2; increase endothelial cell density; prevent necrosis and breakdown of diabetic tissues	^121^
PEMF	Commercially available device (BTL‐4000) with frequency at 25 Hz and intensity of 2 mT or 10 mT	Whole‐body exposure	1 h /day, 3, 5, 7, 10, 14 or 21 days	Male Sprague‐Dawley rats, 10‐week‐old, 300‐400 g	STZ at 50 mg/kg body weight, 1 week after STZ injection	A square full‐thickness wound (6 mm × 6 mm) on the lateral side of each hindlimb	No change on body weight and blood glucose	Improve biochemical properties, increase energy absorption capacity	^122^
PEMF	Commercially available device (XKC‐600 W) with frequency at 25 Hz, intensity of 5 mT and pulse width of 40 ms	Whole‐body exposure	1 h /day, 7, 10 or 14 days	Male Sprague‐Dawley rats, 300‐400 g	STZ at 50 mg/kg body weight, 1 week after STZ injection	A square full‐thickness wound (2 cm × 2 cm) at the back covered with sterile gauze	No change on body weight and blood glucose	Increase Type Ⅰ collagen fibre deposition	^123^
PEMF	Commercially available device XKC‐600W with frequency at 25 Hz, intensity of 5 mT and pulse width of 0.04 ms	Whole‐body exposure	1 h /day, 7, 10, 14 or 21 days	Male Sprague‐Dawley rats, 8 ~ 10 weeks, 280‐320 g	STZ at 50 mg/kg body weight, 72 h after STZ injection	A square full‐thickness wound (2 cm × 2 cm) on the dorsum covered with sterile gauze	No change on body weight and blood glucose	Smaller wound area; greater percentage of healing; significant epidermal gap; more myofibroblasts in the granulation tissue	^124^
PEMF	Commercially available device (FG‐330) with frequency at 20 Hz, intensity of 8 mT and pulse width of 4 ms	Whole‐body exposure	1 h/day, 4, 8 12 or 16 days	Male Wistar rats, 4 months old, 200‐300 g	STZ at 65 mg/kg body weight, 2 weeks after STZ injection	A full‐thickness incision (35 mm in length) on the right dorsum	No change on body weight and blood glucose	Reduce healing time; increase healing rate; increase tensile strength	^125^

**TABLE 3:** The effects of MFs on DFUs based on clinical trials

MF types	Intensity / frequency	Exposure manner	Exposure duration	Patients enrolled	Treatment group	Main findings	Ref
PEMF	Frequency at 12 Hz and intensity of 12 G	Local exposure	60 min/session, 14 session in 3 weeks	13 patients diagnosed with type 2 diabetes with unsatisfactory healing of ulcer in the preceding 4 weeks	Two groups with application of PEMF or not	18% decrease in wound size in PEMF group; significant cumulative increase in cutaneous capillary blood velocity and capillary in diameter.	^135^
ELF‐EMF	120 Hz and 0.4‐0.9 mT RMS	Local exposure	Forearm: 2 h/day, twice weekly; thorax: 25 min/day, twice weekly	26 diabetic patients with non‐responsive DFUs	All receive ELF‐MF	No adverse effects or ulcer recurrences in the original ulcer site	^136^
PRFM	ActiPatchTM (BioElectronics Corporation, Frederick, MD) which delivers PRFE at a carrier frequency of 27.12 MHz and a pulse rate of 1000 Hz	Local exposure	6‐8 h per day for 6 consecutive weeks	4 patients with diabetic ulcers present for longer than 3 months	All receive PRFM	After 1 week, reduced wound size of ulcers which presenting in diabetic patients for more than 3 months, two patients come to complete healing of the ulcers with 3 weeks of treatment	^138^
PEMF	TMR‐9 equipment (THERESON, Milano, Italy)	Local exposure	2 consecutive weeks, 20 min each, twice a day	20 patients with diabetes mellitus lasting more than 5 years and having active ulcerated DFU	Two groups with application of TMR or not	Increase healing rate and healing time at 6 months, increase rate of granulation tissue at 3 months, without any significant adverse event	^139^
PEMF	Therapeutic Magnetic Resonance (TMR®) (Thereson Srl, Vimercate, MB, Italy) 40‐60 µT	Local exposure	2 consecutive weeks	40 patients with type 2 diabetes lasting for 5 years or more; having a distal neuropathic ulcer to the foot started more than 6 weeks larger than 1 cm^2^	Two groups with application of TMR or not	More lesions healed, faster healing time, improve the quality of granulation tissue, enhanced differentiated keratinocytes, more deposition of collagen fibres, higher expressions of collagens, intergrin alpha 1, intergrin beta 3, MMPs, cytoskeleton proteins, interleukin and lesser expression of pro‐inflammatory cytokines	^140^

### 4.1 The effects of MFs on diabetic wound healing from animal experiments

180 mT gradient SMF with the North pole orienting towards the wound promotes the development of capillaries, increases the healing rate and reduces the gross healing time in streptozotocin (STZ)‐induced diabetic rats with an open circular wound in the dorsum.^118^ SMFs exhibit different effects at different wound healing phases.^119^ SMF exposure decreases the number of inflammatory cells and necrosis level at the wound site in the early stage of wound healing in diabetic rats; furthermore, SMFs treatment activates the re‐epithelialization process and the development of the capillaries in the middle wound healing stage; finally, SMFs promote organized deposition of mature collagen fibres at the wound sites. SMFs also facilitate the transition of wound healing phases in diabetic conditions. 0.6 T SMF accelerates wound closure and elevates re‐epithelialization and revascularization in diabetic mice by skewing macrophage polarization towards M2 phenotype and upregulating the anti‐inflammatory signalling. In a diabetic rat model, the wound healing effect of 230 mT SMF is demonstrated by evaluating the wound area reduction rate, the mean time to wound closure and the wound tensile strength.^120^

Several studies show that dynamic MFs improve the various stages of wound healing, but they play different roles. PEMF generating from a commercially available bone‐healing device improves wound closure rate by increasing fibroblast growth factor 2 (FGF‐2) and endothelial cell density in diabetic mice and prevents necrosis and breakdown of diabetic tissues.^121^ Biomechanical properties of wounds are mainly decided by the amount of collagen, fibril alignment and fibre orientation and reflect the structural recovery of wounds and function. PEMF with frequency at 25 Hz and intensity of 10 mT improves the tensile biomechanical properties associated with increased maximum load and energy absorption capacity in the early diabetic wound healing phase, but in the remodelling phase, it weakens the wounds possibly through the prolonged collagen deposition.^122^ Another study also supports that PEMF exhibits different effects at the different phases of diabetic wound healing. After exposure to a commercially available PEMF unit, there is greater abundance of collagen fibre and enhancement of myofibroblasts in the early phase of diabetic wound healing, while the alignment and orientation of collagen fibril seem no change.^123^ PEMF enhances wound closure and re‐epithelialization with production of myofibroblasts which play a key role in wound closure and collagen synthesis in wound healing process.^124^ In an animal study, diabetic rats exposed to LF‐PEMF show reduced time of wound healing and increased tensile strength of scar.^125^

### 4.2 The effects of MFs on diabetic foot ulcers from clinical trials

DFUs, one of the most common and severe complications of DM, are characterized with severely impaired wound healing.^126^, ^127^ The aetiology of DFU is multifactorial and classified into neuropathic, ischaemic and neuro‐ischaemic ulcers.^128^, ^129^ Diabetic people with DFUs are always associated with the prevalence of chronic vascular diabetic complications.^130^ Peripheral vascular disease, peripheral neuropathy which is caused by microvascular complications in DM and peripheral arterial disease which is caused by macrovascular complications in DM are risk factors for contributing to DFUs.^131^ Thus, in view of the complexity of origins of DFU, it is of great importance to understand the differences and healing process in these types of DFUs for effective prevention and management.

The physical interventions to improve the healing outcomes of DFUs include negative pressure, electrical fields, lasers, ultrasound, shockwaves and dynamic MFs.^132^, ^133^, ^134^ Accumulative studies from cellular level and animal models investigate the effects of MFs on wound healing process and demonstrate their positive effects. There are also commercially available MFs devices developed for clinical applications on wound healing. Trials that apply MFs on DFUs management are summarized in Table 3.

In a randomized, double‐blind and placebo‐controlled clinical trial, patients who receive PEMF therapy with frequency of 12 Hz and intensity of 12 G for 60 min during one session show 18% decrease in wound size, 14% increase in capillary diameters, 28% increase in cutaneous capillary blood velocity and 16% increase in skin blood flow, whereas 10% decrease in wound size in the sham MF group after 14 sessions within 3 weeks.^135^ With forearm and thorax exposure to ELF‐MF, a clinical phase 2 study through a long‐term follow‐up shows that there are no adverse effects or ulcer recurrences at the original ulcer sites in DFU patients.^136^ However, there is no sham ELF‐EMF treated group, it is hard to evaluate the effectiveness of ELF‐EMF on DFUs through this trial. Technologic advances allow the development of EMF device which are portable for daily use.^137^ In a case report, four patients using pulsed radio frequency electromagnetic (PRFE) wearable device for 6‐8 hours per day for consecutive 6 weeks show promising results in reducing the size of foot ulcers which have been presented in these patients for more than 3 months, among them, two patients come to complete healing with 3 weeks of treatment.^138^ Therapeutic magnetic resonance (TMR) can generate low‐intensity MFs. In a pilot trial, when diabetic patients with foot ulcers are treated with TMR, the outcome shows that there is an increase in healing rate after 6 months treatment.^139^ In addition, this portable magnetic device is also used in another clinical trial to investigate its effects on DFUs. After receiving daily home therapy with TMR device, diabetic patients with ulcers show more healed lesions with well‐organized cells into the epidermal and dermal tissue, enhanced differentiated keratinocytes, more deposition of collagen fibres, improved quality of granulation tissue and faster healing time.^140^ The normal wound healing process is mediated by various cytokines, and similarly, they play indispensable role in the management and care of DFUs.^141^ Study has revealed that the impaired formation of granulation tissue stalls wound healing at inflammation phase in non‐healing DFUs.^142^ When applying TMR treatment, histological and biological examinations further show significant signs of wound healing with higher expressions of collagens, integrin α1, integrin β3, MMPs, cytoskeleton proteins, anti‐inflammatory interleukin, growth factors including FGF, FDGF and vascular endothelial growth factor (VEGF), and a lower expression of pro‐inflammatory cytokines.^140^

So far, the reported clinical trials by using MFs to intervene DFUs show no adverse events during or at the end of the treatment or through the follow‐up investigation. Although MFs have been applied for treating DFUs in several trials, the evidence of MFs’ benefit effects cannot be fully ruled out as for the shortcoming of the small number of patients included, the multiple assessment methods, the different MF device used, the diverse range of treating time and the inadequate trial groups.^143^ Moreover, there is no aetiology classification of enrolled diabetic patients with foot ulcers in these clinical trials, it is hard to explore the potential effectiveness and action of mechanism of magnetic fields on different origins of ulcers. The correct and effective approach to treat DFUs may directly influence the clinical outcome.

## 5 FACTORS AFFECT THE OUTCOMES OF MAGNETIC FIELDS ON DIABETIC WOUNDS

**FIGURE 2:**
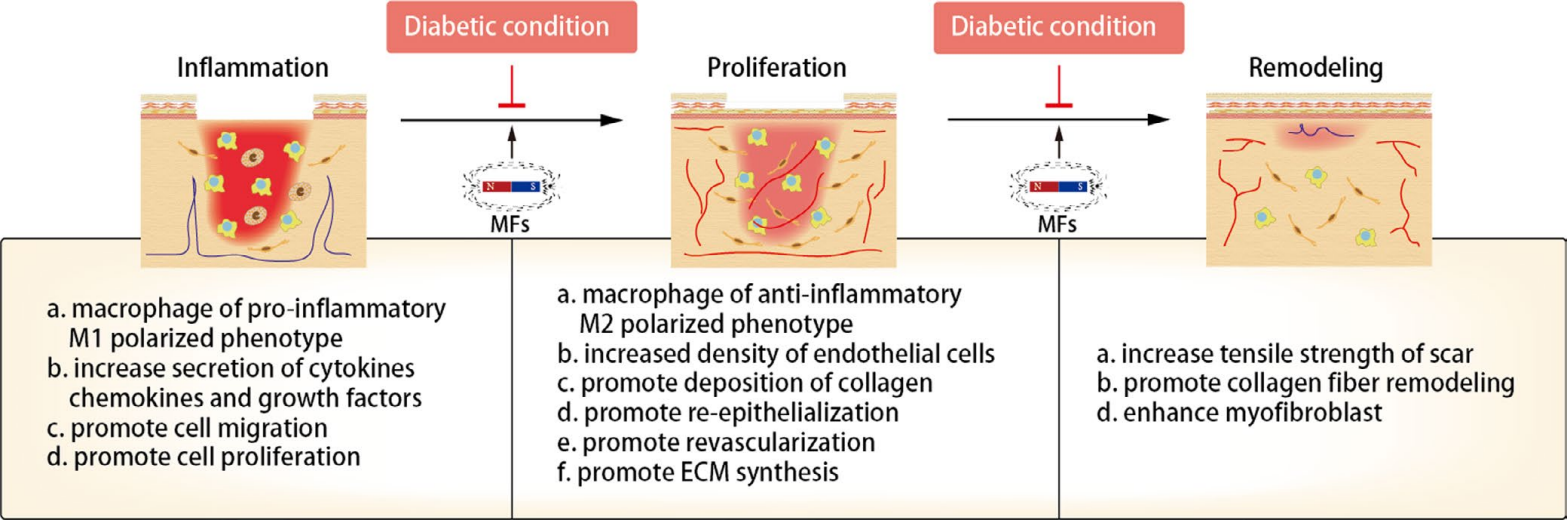
The possible mechanism of the effects of MFs on diabetic wound healing. Abbreviations: ECM, extracellular matrix

Although there are ample studies carried out to support the positive effects of MFs on wound healing in DM either from cellular level or animal models, the effects and application of MFs on wound healing in clinical trials are still poorly demonstrated. The action of MFs on diabetic wounds is a complex interaction between physical factor and living organism (Figure 2). Some factors should be considered when it comes to evaluating the bio‐effects and the potential therapeutic effects of MFs on diabetic wounds.

In this review, factors that may explain the discrepancy in MFs’ effectiveness in diabetic wound healing are concluded as following. First, the constructed and used diabetic wound models should be considered. As seen in Table 1, several types of diabetic animal models have been used to evaluate the effects of MFs on diabetic wounds. Some studies use chemical‐induced diabetic condition, for example STZ or alloxan, while others use genetic diabetic animals even with different animal strains. Some studies constructed wounds with small size while others use large ones. Diabetic animal models of impaired wound healing may lead many to question MFs’ effectiveness.

The next factor is the characteristics of MFs applied. Physical parameters and patterns of MFs affect their bio‐effects. As to SMF, the difference in field intensity and direction show obvious difference in affecting wound healing. With regard to dynamic MFs, it is even more complex for the differences between intensity, frequency, pulsed width, duration and exposure frequency from dynamic MFs generating devices.

**FIGURE 3:**
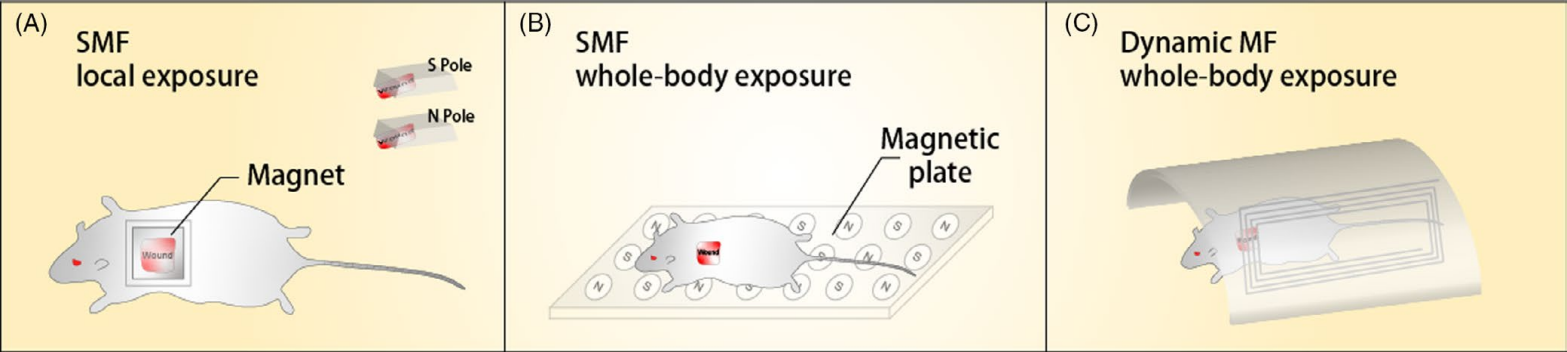
The exposure manner of MFs can be classified into local and whole‐body manner. Abbreviations: SMF, static magnetic field; Dynamic MF, dynamic magnetic field

The third factor is the exposure manner of MFs (Figure 3). It can be classified into local exposure and whole‐body exposure. SMFs generated from permanent magnets are easily used to directly place near the wound sites, and it is also possible to achieve whole‐body exposure. The diabetic wounded animals are exposed to dynamic MFs whole‐body, it is hard to investigate whether the positive effects on wound healing are ascribed to their effects on wound sites or the regulation on the whole body. The effects of MF exposure manners make the mechanism involving in diabetic wound healing even more complex to explain.

The bio‐effects of MFs are largely dependent on the stimulation time, so the forth factor is the duration of MFs exposure. Wound healing is a complex process that matters of time. It is important to choose the suitable duration and frequency of MFs exposure and in which wound healing stage MFs exposure can work best. As to dynamic MFs exposure, the tissues experience a heated process, the short duration of dynamic MFs exposure may protect the biological tissues from development of increased temperature.

As far as we known from Tables 1, 2, 3, dynamic MFs are used in clinical trials to intervene DFUs, while there is no related report about the application of SMF in clinical trials. In laboratory‐based experiments for intervening DFUs, magnets are usually adopted to generate SMF and the field strength is lower and limited to only hundreds mT. The interaction outcomes of SMF with living organisms are mild and closely related to intensity and largely dependent on exposure time. The field strength of a magnet is easy to remain stable. But the stronger the magnetic field intensity, the heavier and bulkier the magnet. Except for MRI, the higher intensity of SMF is rarely used in clinical therapy for the inconvenient and possibly using superconductive technology along with the expensive operating and maintenance costs. As to dynamic MFs, they have been widely developed and used in clinics for treating bone and neurodegenerative diseases. The actions of dynamic MFs on living organisms are fast and instantaneous with high efficiency through the interactions of electromagnetic energy and force with biomolecules with electromagnetic properties. However, the safety issues of dynamic MFs exposure are still controversial.

## 6 CONCLUSIONS

To summarize, MF as a kind of noninvasive and safe physical therapeutic approach has been shown great potential of application prospects in diabetic wound healing with no significant side effects. Although a majority of studies have indicated the positive effects of MFs on diabetic wounds, there is still no general agreement on the exact mechanisms related to such biological or therapeutic effects, and there remain many unknown aspects to focus on. It is also encouraged to develop more domestically portable equipment generating MFs to manage chronic wounds for people with diabetes at home, and push forward more accessible usage of physical therapy to reduce both the mental and financial burden in people with diabetes.

## CONFLICT OF INTEREST

None declared.

## AUTHOR CONTRIBUTIONS

HH Lv wrote the manuscript. JY Liu drew the figures. CX Zhen, YJ Wang, YP Wei and WH Ren revised the manuscript. P Shang supervised the manuscript. All authors read and approved the final version of the paper.

## Data Availability

The data that support the conclusions of this work are available from the first author and the corresponding author.
